# The role of the fronto-parietal network in modulating sustained attention under sleep deprivation: an functional magnetic resonance imaging study

**DOI:** 10.3389/fpsyt.2023.1289300

**Published:** 2023-11-16

**Authors:** Linming Yao, Yajing Wang, Yanzhong Gao, Hongwei Gao, Xufeng Guo

**Affiliations:** ^1^The Tuberculosis Control and Prevention Hospital of Shaanxi Province (The Fifth People’s Hospital of Shaanxi Province), Xi’an, China; ^2^Department of Orthopaedics, Xi’an No. 9 Hospital, Xi’an, China

**Keywords:** sleep deprivation, fronto-parietal network, fractional amplitude of low-frequency fluctuations, psychomotor vigilance task, dorsolateral prefrontal cortex

## Abstract

**Objective:**

The intricate relationship between sleep deprivation (SD) and cognitive performance has long been a subject of research. Our study offers a novel angle by closely examining the neurobiological underpinnings of sustained attention deficits through the lens of the fronto-parietal network (FPN). Using state-of-the-art imaging techniques, we delve into the changes in spontaneous brain activity after SD and explore their associations with performance on the psychomotor vigilance task (PVT).

**Methods:**

We conducted an elaborate investigation involving 64 healthy, right-handed participants who underwent resting-state functional MRI scans before and after experiencing 24 h of sleep deprivation. Employing sophisticated statistical analyses, we scrutinized the changes in fractional amplitude of low-frequency fluctuations (fALFF) through paired *t*-tests. Pearson correlation analyses were then applied to dissect the associations between these neurobiological shifts and behavioral outcomes in PVT.

**Results:**

The study yielded remarkable findings, revealing a dramatic decrease in fALFF values within critical areas of the FPN following SD. These alterations predominantly occurred in the frontal and parietal gyri and were inversely correlated with PVT performance metrics. Furthermore, we discovered that baseline fALFF values in the left dorsolateral prefrontal cortex (DLPFC) have the potential to serve as compelling neurobiological markers, with high discriminatory power in identifying individual responses to the adverse effects of SD on cognitive performance.

**Conclusion:**

Our groundbreaking research underscores the pivotal role that the FPN plays in modulating attention and executive function, especially under the challenging conditions brought about by sleep deprivation. The findings offer critical insights that could shape the way we understand, assess, and potentially mitigate the cognitive impacts of SD, setting the stage for future research in this riveting domain.

## Introduction

Sleep deprivation (SD) is a widespread issue affecting various professional sectors, including transportation, healthcare, and the military (1). Acute total SD has been shown to detrimentally impact multiple cognitive domains such as working memory, decision-making, motor control skills, and attention. Notably, sustained attention, a critical cognitive function, involves the consistent detection of rare and unpredictable signals over extended periods, playing a significant role in various daily activities and professional tasks (2) previous studies have indicated that sustained attention suffers most significantly from SD (3). Slower reaction times and increased lapses were commonly observed after SD (4). Understanding the neural mechanisms underlying the impact of SD on sustained attention can inform strategies for mitigating these effects, with substantial implications for both individual well-being and public safety. Subsequent sentences discussing specific neuroimaging studies.

Numerous neuroimaging studies have demonstrated that sustained attention impairments after sleep deprivation are associated with multiple brain functional alterations. The first psychomotor vigilance task (PVT)-related functional magnetic resonance imaging (fMRI) study demonstrated that the activation strengths within the inferior parietal lobes, and the right dorsolateral prefrontal cortex were compromised after SD (4). A comprehensive review by Basner et al. (5) indicated diminished activity in the intraparietal sulcus, right prefrontal cortex, and medial frontal cortex following SD. Subsequent research has consistently observed reduced activation in the dorsolateral prefrontal cortex and intraparietal sulcus during attention tasks after SD (6, 7). Moreover, a recent task-fMRI study employing five PVT-fMRI observation sessions during SD identified decreased activation in regions such as the bilateral middle frontal gyrus, right inferior frontal gyrus, right supplementary motor area, left superior parietal gyrus, left inferior, and left paracentral lobule during late-night periods of SD (8). Collectively, these findings suggest that impaired sustained attention following SD may be attributable to diminished activity within the frontoparietal cortex.

Additionally, substantial inter-individual variability exists in the susceptibility to sustained attention impairments following SD. This vulnerability has been shown to be stable, replicable, and trait-like in nature (9, 10). Research by Chee et al. (7) has demonstrated that individuals resistant to SD exhibit higher activation levels within the frontoparietal network during sustained attention tasks as compared to those who are more vulnerable. Weissman et al. (11) further identified that lapses in vigilance are often preceded by decreased activity in the prefrontal cortex and parietal gyrus, implying a disruption in top-down control mechanisms governing attention. Optimal performance in sustained attention tasks has been correlated with elevated activation within the frontoparietal network, suggesting that this network may play a role in the top-down modulation of attention required for task execution (4). Consequently, baseline spontaneous activity within the frontoparietal network may offer a promising avenue for differentiating between SD-resistant and SD-vulnerable individuals.

In recent years, resting-state functional magnetic resonance imaging (rs-fMRI) has emerged as a particularly valuable tool in this realm due to its non-invasive nature and sensitivity to neurophysiological changes (12). This technique stands out in its capacity to unveil the brain’s intrinsic functional connectivity—neural activity patterns occurring during rest—providing critical insights into the baseline functional connectivity alterations induced by sleep deprivation. Furthermore, fractional amplitude of low-frequency fluctuations (fALFF) analysis (13), by quantifying the amplitude of low-frequency fluctuations, effectively captures regional brain activity intensity, presenting a meaningful way to correlate these intrinsic activity changes with cognitive deficits observed following sleep deprivation. When compared to other neuroimaging methods, rs-fMRI, particularly in combination with fALFF analysis, holds distinct advantages including reduced performance variability and a more nuanced interpretation of the resting brain’s physiological state, especially pertinent in the context of sleep deprivation research. In the present study, we employ rs-fMRI-derived fALFFto explore changes in spontaneous neural activity within the fronto-parietal network (FPN) following 24 h of sleep deprivation (SD). Furthermore, we assess the association between these neural changes and impairments in sustained attention as measured by the psychomotor vigilance task. Our study also aims to investigate whether baseline fALFF values within the FPN can serve as predictive markers to differentiate between individuals who are resistant to SD-induced cognitive impairments and those who are vulnerable.

## Materials and methods

### Participants

This study was conducted in compliance with the ethical guidelines outlined in the Declaration of Helsinki and received approval from the Ethics Committee of Xi’an No. 9 Hospital. Written informed consent was obtained from each participant prior to their involvement in the study. Participants were recruited from the local community through targeted advertisements. Eligibility criteria for inclusion in the study were as follows: (1) right-handed, (2) between 18–65 years old. The exclusion criteria were: (1) presence or history of medical disease, (2) presence or history of sleep disorders, (3) presence or history of psychiatric diseases, (4) shift workers, (5) a history of substance abuse or dependence and (6) any contraindication to MRI scanning.

### Experiment paradigm

Participants underwent two separate MRI scanning sessions: one following a Resting Wakefulness (RW) period and another after SD. A minimum interval of 1 week between sessions was maintained to mitigate any lingering effects of SD on both cognitive function and fMRI signals. For the SD session, the process commenced at 8:00 AM on the first day and concluded at 8:00 AM the following day. During this 24 h period, participants were mandated to remain awake and were continuously monitored by research assistants to ensure compliance. For the RW session, participants arrived at the laboratory at 7:00 AM after a normal night’s sleep, having rested for a minimum of 8 h. Prior to each MRI scan, all participants completed a 10 min Psychomotor Vigilance Task to assess sustained attention performance. The PVT was chosen because it provides a direct, objective, and reliable measure of sustained attention, separate from other cognitive functions. Its simplicity, real-world relevance, and adaptability make it particularly useful for research into the factors that can impair or enhance human alertness and attentional performance (14). During the test, red dots were displayed on a screen, and participants were instructed to respond as rapidly as possible by pressing a designated key on the keyboard when the red dots appeared. These visual stimuli persisted for 1,000 ms and were immediately removed upon the participant’s response. Response times (RT) were recorded for each stimulus, with RTs exceeding 500 ms classified as lapses. Finally, for each participant, the mean RT, the mean 20% fastest RT, the mean 20% slowest RT and the mean number of lapses were calculated. Following the completion of the PVT, participants were immediately escorted to the MRI facility, where the scanning commenced without delay, ensuring that the neural data captured reflected the cognitive state post-PVT.

### MRI data collection

All MRI data were collected using a 3.0 Tesla GE Discovery 750 scanner (General Electric, Milwaukee, Wisconsin). Participants were instructed to remain motionless and awake throughout the scanning sessions. Functional images during resting-state were captured using a gradient-echo planar imaging sequence. Parameters were set as follows: field of view (FOV) 240 × 240 mm, data matrix 64 × 64, echo time (TE) 30 ms, repetition time (TR) 2,000 ms, with 33 slices. A total of 210 images were acquired for each participant. For anatomical referencing, T1-weighted images were obtained using a Bravo sequence. The scan settings included a repetition time of 8.2 ms, an echo time of 3.2 ms, a FOV of 256 × 256 mm, a data matrix of 128 × 128, a slice thickness of 1 mm, and 196 axial slices.

### fMRI data preprocessing

The preprocessing of resting-state fMRI images was conducted using the Data Processing & Analysis for Brain Imaging (DPABI) software package, which integrates procedures from the Resting State fMRI Data Analysis Toolkit (REST) and Statistical Parametric Mapping (SPM12). After discarding the first 10 images to reach magnetization equilibrium, the remaining 200 images underwent slice timing correction and motion realignment. Mean frame-wise displacement (FD) was calculated, and participants exhibiting greater than 2 mm maximal translation or 2° maximal rotation were excluded from further analysis. We employed the Friston-24 model to regress out head motion effects and nuisance signals from the cerebrospinal fluid and white matter. Subsequently, the preprocessed data were spatially normalized into the Montreal Neurological Institute (MNI) space using the diffeomorphic anatomical registration through exponentiated lie algebra (DARTEL) technique. Post-normalization, the images were smoothed with a 6 mm full-width at half-maximum Gaussian kernel and underwent band-pass filtering in the range of 0.01–0.08 Hz.

For calculating fractional amplitude of low-frequency fluctuations (fALFF), each voxel’s time series was transformed into the frequency domain. The power spectrum within the 0.01–0.08 Hz frequency band was then squared to compute the amplitude of low-frequency fluctuations (ALFF). After summing ALFF values across the specified frequency range, fALFF values were generated by normalizing each voxel’s ALFF by the sum of ALFF values across the entire frequency band.

### Statistical analysis

**Figure 1:**
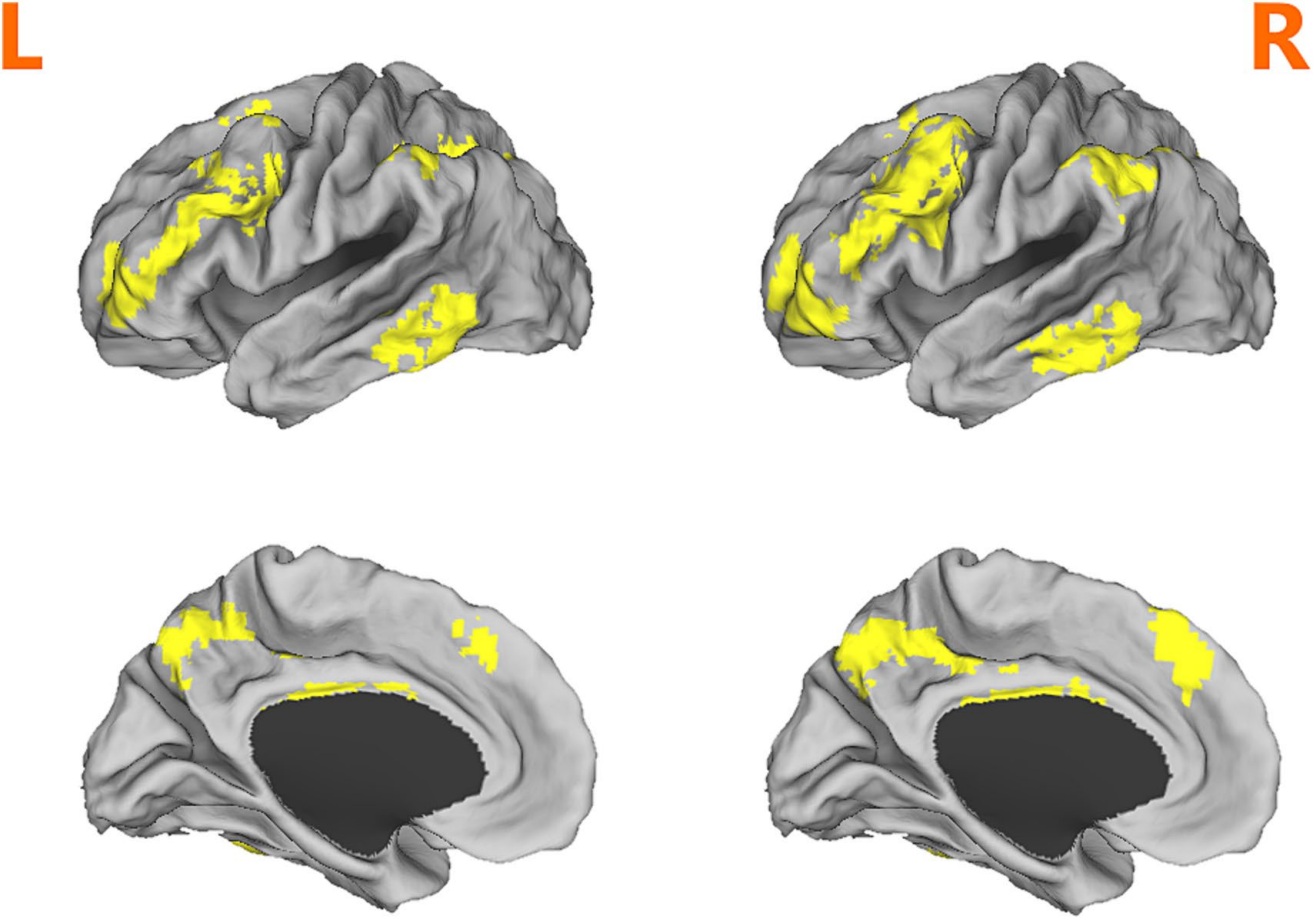
Depiction of the fronto-parietal network mask used in analyses. Adapted from (15).

Paired *t*-tests were conducted to identify changes in fALFF between the RW and SD conditions. All analyses were limited to regions within the fronto-parietal network mask [see Figure 1 for the mask (15)], mean frame-wise displacement calculated during preprocessing was included as a covariate. Pearson correlations were utilized to examine the relationship between fALFF changes and PVT performance indicators, specifically mean RT, fastest 20% RT, slowest 20% RT, and the mean number of lapses. These analyses were conducted using IBM SPSS Statistics for Windows (version 18.0), with a Bonferroni-corrected significance level set at *p* < 0.05. Then, Participants were categorized into SD-resistant and SD-vulnerable groups based on PVT lapse changes between RW and SD conditions. Out of the total cohort, 32 participants were classified as SD-vulnerable, exhibiting significant performance decrements during the PVT post-sleep deprivation, while another 32 were deemed SD-resilient, demonstrating minimal performance changes despite the lack of sleep. Two-sample *t*-tests examined fALFF baseline differences between these groups, with corrections for multiple comparisons made using the false discovery rate (FDR) criterion. Finally, a receiver operating characteristic (ROC) curve was employed to assess the potential of baseline fALFF values as neurobiological indicators capable of distinguishing between SD-resistant and SD-vulnerable groups. Crucially, we assessed the area under the ROC curve (AUC), providing a comprehensive measure of our model’s overall discriminative ability in the context of attentional performance deficits post-sleep deprivation.

## Results

### Demographic features and PVT performance

In total, 64 right-handed healthy participants were included in the study. Sleep diaries and actigraphy data confirmed normal sleep patterns across the sample (mean ± standard deviation: 7.45 ± 1.38 h of sleep per night). No significant differences in sleep metrics were observed between the SD-resilient and SD-vulnerable subgroups (*t* = 0.14, *p* = 0.89). For the PVT performance, overall slower reactions were found after SD, paired-*t*-test indicated that the mean RT (*t* = −6.25, *p* < 0.001), the mean RT of the 20% fastest trails (*t* = −5.63, *p* < 0.001), the mean RT of the 20% slowest trails (*t* = −4.93, *p* < 0.001), and the number of the lapse (*t* = −8.98, *p* < 0.001) were significantly increased after SD. Based on the change of PVT lapse, participants were divided into SD-vulnerable and SD-resilient groups. Notably, significant differences (*t* = 13.09, *p* < 0.001) was found between the SD-vulnerable (10.65 ± 3.21) and SD-resilient (1.47 ± 2.32) groups. Further details are presented in Table 1.

**Table 1:** Demographic characteristics, objective sleep measures and PVT performance.

**Demographic information**	
Gender (male/female)	34/30
Age (years)	23.17 ± 1.87
Body mass index	22.34 ± 1.26
**Objective sleep from Actiwatch**	
Number of wakening	24.22 ± 7.82
Sleep duration all night	7.55 ± 1.28
Sleep efficiency in %	85.17 ± 3.08
Sleep latency in minutes	21.37 ± 8.56

PVT performance	RW	SD	*t*-value	*p*-value
Mean RT (ms)	346.72 ± 434.59	486.27 ± 177.42	−6.25^a^	<0.001
Mean fastest RT (ms)	280.57 ± 24.95	305.73 ± 31.86	−5.63^a^	<0.001
Mean slowest RT (ms)	437.69 ± 54.41	897.73 ± 746.97	−4.93^a^	<0.001
Number of lapse	1.83 ± 1.28	7.88 ± 5.30	−8.98^a^	<0.001

a *t*-value obtained by using the paired *t*-test.

### Changes in fALFF within the FPN network after SD

**Figure 2:**
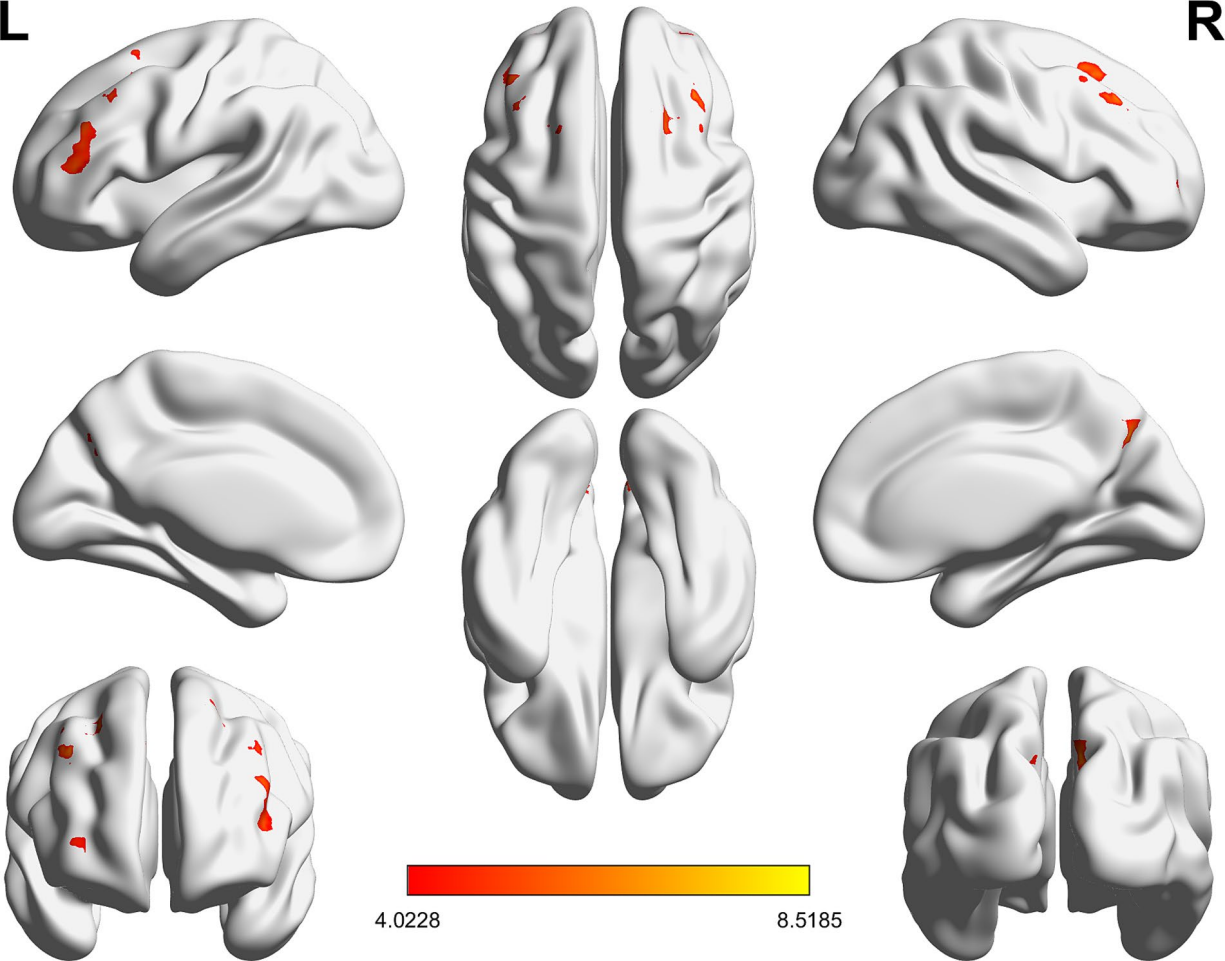
Brain regions within the frontoparietal network (FPN) displaying reduced fALFF values following sleep deprivation (SD) compared to resting wakefulness (RW).

**Figure 3:**
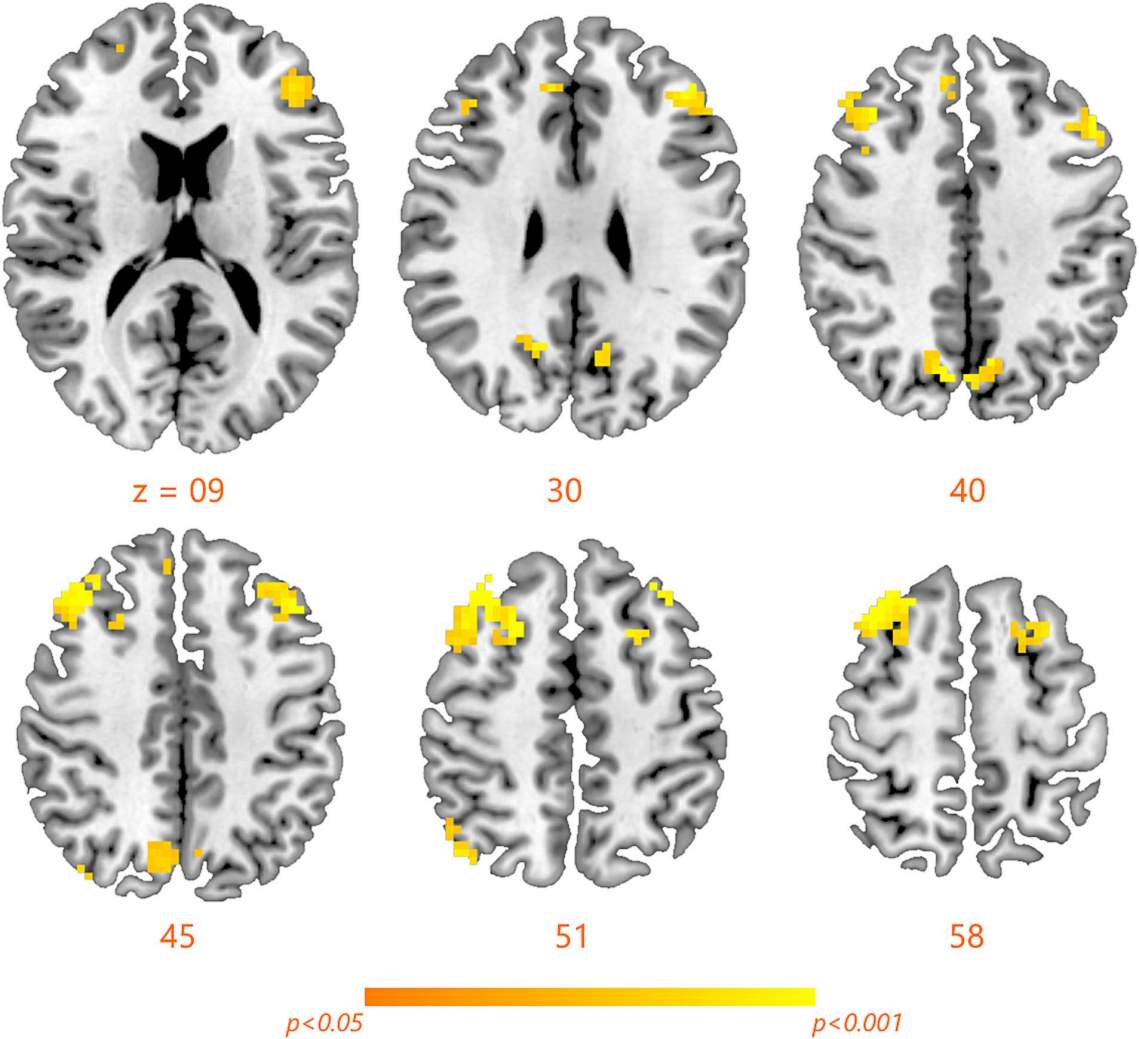
Detailed maps highlighting areas of decreased spontaneous activity in the bilateral middle frontal gyrus, bilateral superior frontal gyrus, left superior parietal gyrus, and right inferior parietal gyrus following SD.

**Figure 4:**
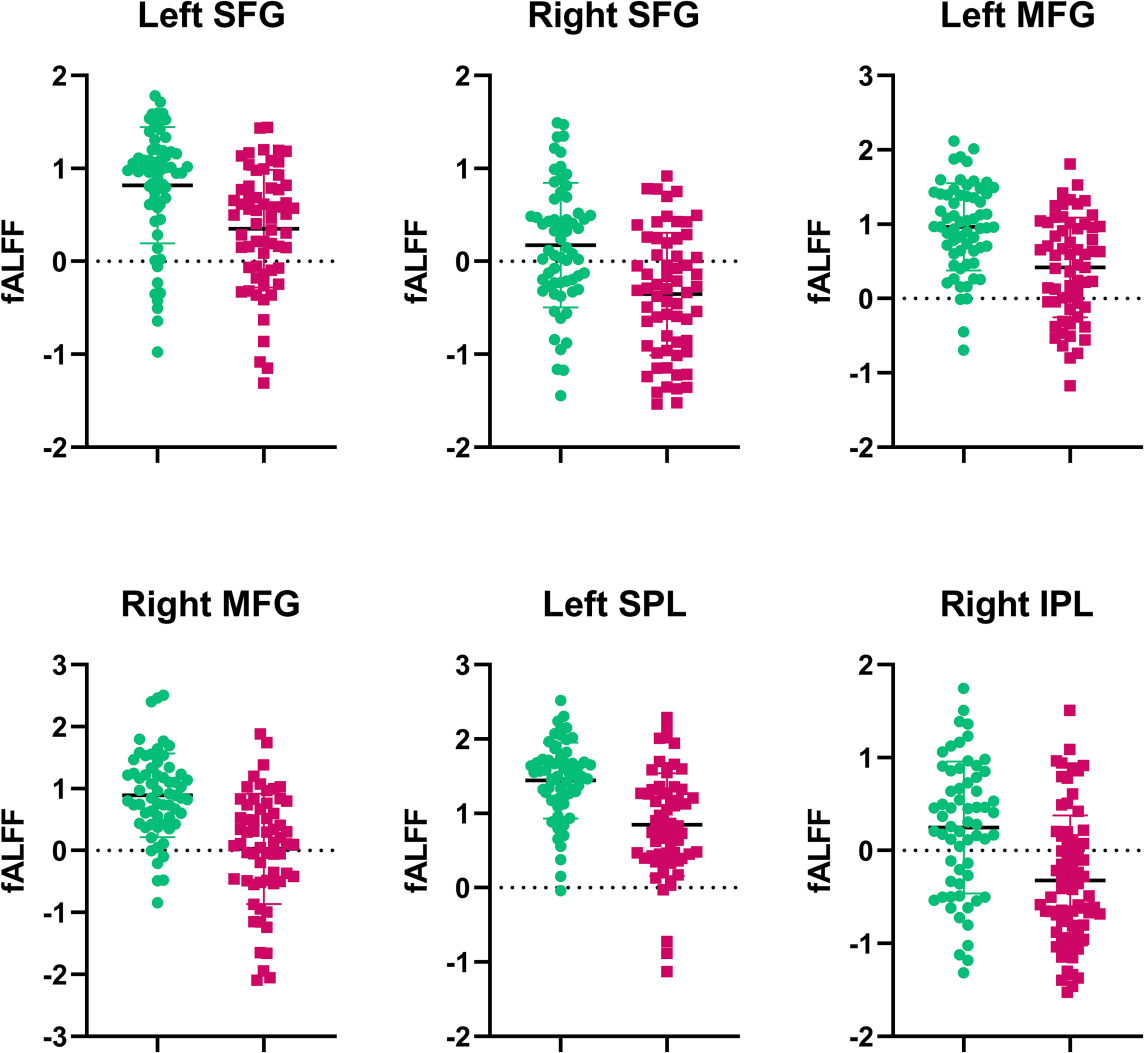
Extracted fALFF values from brain regions with significant changes post-SD. Each bar represents the average fALFF value for the respective region.

Compared with RW condition following a normal sleep, significantly reduced fALFF values were found within the FPN network. Specifically, the decreased spontaneous activities were mainly found in bilateral middle frontal gyrus, bilateral superior frontal gyrus, the left superior parietal gyrus and the right inferior parietal gyrus. These findings are graphically represented in Figures 2, 3 and tabulated in Table 2. offers a more nuanced view of the changes in spontaneous brain activity after SD, we also extracted the fALFF values for these regions and present them in Figure 4.

**Table 2:** Significantly reduced fALFF after sleep deprivation.

Baseline < HC	Voxels	Peak coordinates (MNI)			*t*-value
		*x*	*y*	*z*	
**Middle frontal gyrus**					
Left	181	−42	24	45	6.64
Right	291	36	27	54	5.21
**Superior frontal gyrus**					
Left	42	−30	54	0	4.84
Right	53	9	39	30	5.37
**Superior parietal lobule**					
Left	152	−9	−69	39	6.92
**Inferior parietal lobule**					
Right	48	9	−30	63	5.45

### Associations between fALFF alterations and PVT metrics changes

**Figure 5:**
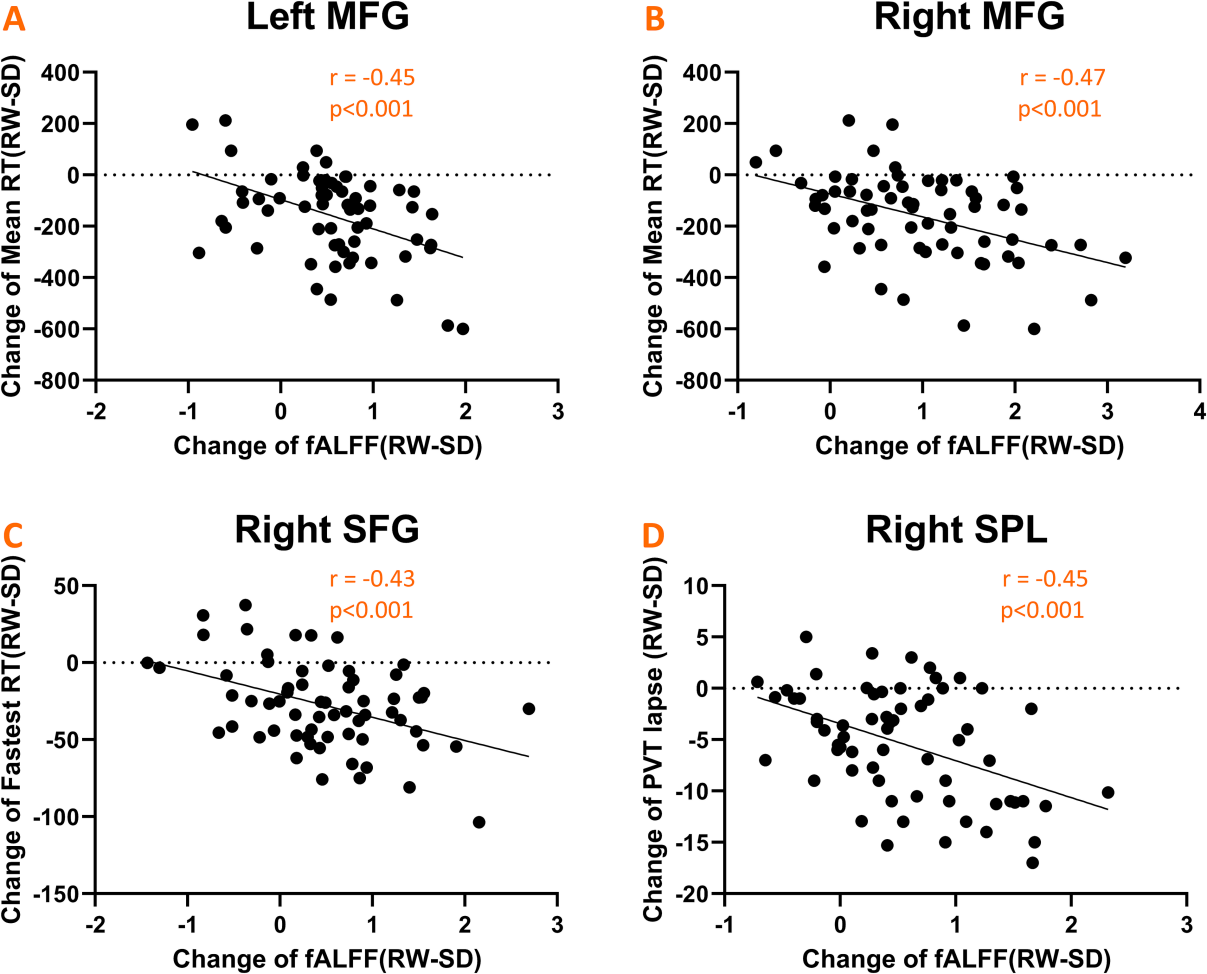
Scatter plots illustrating significant negative correlations between changes in fALFF values and psychomotor vigilance task (PVT) performance metrics. **(A,B)** Shows correlations with mean RT in the bilateral middle frontal gyrus; **(C)** shows correlation with fastest RT in the left superior frontal gyrus; **(D)** shows correlation with the number of lapses in the right superior parietal gyrus.

We explored the link between fluctuations in fALFF values and changes in PVT performance. Notable negative correlations emerged, as depicted in Figure 5. Specifically, alterations in mean RT were inversely correlated with changes in fALFF within the bilateral middle frontal gyrus (*r* = −0.45, *p* < 0.001; Figure 5A) and (*r* = −0.47, *p* < 0.001; Figure 5B). Similarly, the fastest RT changes were inversely related to fALFF values in the left superior frontal gyrus (*r* = −0.43, *p* < 0.001; Figure 5C), while changes in the number of lapses negatively correlated with fALFF alterations in the right superior parietal gyrus (*r* = −0.45, *p* < 0.001; Figure 5D).

### Influence of baseline activity on sleep deprivation vulnerability

**Figure 6:**
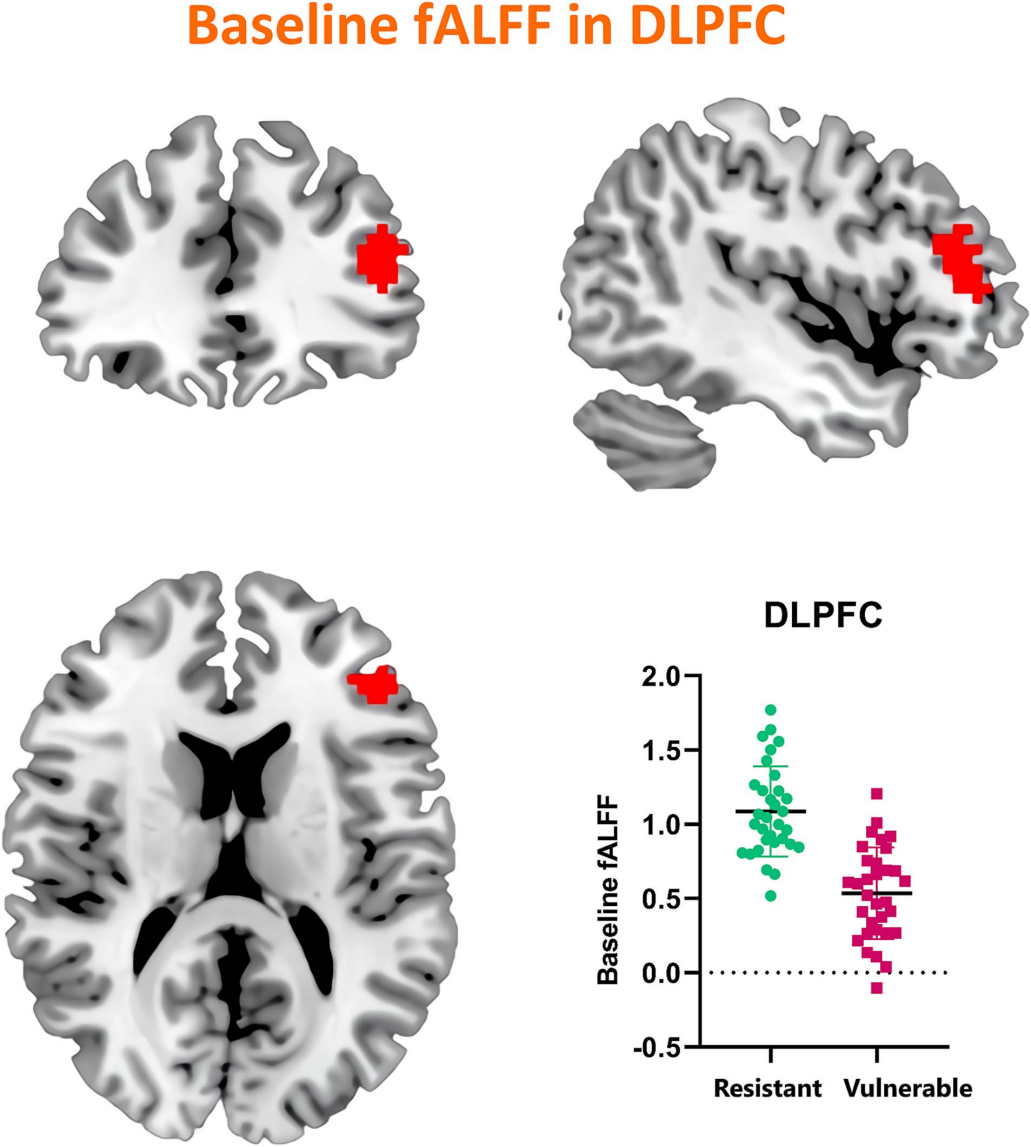
Comparative analysis of baseline (RW) spontaneous activity levels in the left DLPFC between SD-resistant and SD-vulnerable individuals.

**Figure 7:**
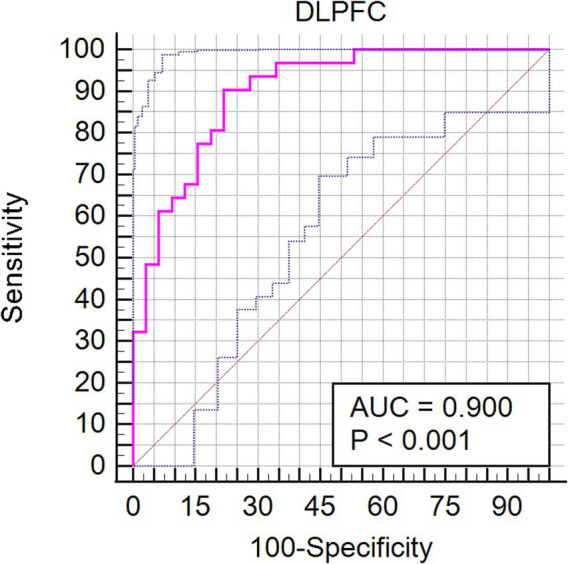
Receiver operating characteristic (ROC) curve showcasing the diagnostic potential of fALFF values within the left DLPFC for differentiating between SD-resistant and SD-vulnerable groups.

Our investigation extended to examining how baseline (RW) neural activity could influence susceptibility to sleep deprivation. Intriguingly, we observed higher spontaneous activity within the left dorsolateral prefrontal cortex (DLPFC) among SD-resistant individuals compared to SD-vulnerable ones (Figure 6). Further, receiver operating characteristic (ROC) analysis revealed that activity levels in the left DLPFC could effectively discriminate between the two groups. Specifically, fALFF values within this region exhibited high discriminatory accuracy, evidenced by an area under the curve (AUC) value of 0.90 ± 0.04 (range 0.80–0.96). Diagnostic tests confirmed high sensitivity (90.62%, CI 75%–98%) and specificity (78.12%, CI 60%–90.7%) for this measure (Figure 7).

## Discussion

This study delved into the influence of sleep deprivation on spontaneous neural activities within the fronto-parietal network. We observed a significant reduction in fALFF values post-SD, primarily localized in the frontal and parietal gyrus. Additionally, we identified robust negative correlations between alterations in PVT performance and fALFF shifts within specific regions of the FPN. These included the mean RT correlating with the bilateral middle frontal gyrus, the fastest mean RT with the left superior frontal gyrus, and the number of lapses with the right superior parietal gyrus. Intriguingly, when assessing the RW condition, we found that the SD-resistant group exhibited higher fALFF values in the left DLPFC compared to the SD-vulnerable group. Furthermore, these baseline fALFF measures demonstrated a high discriminatory capacity for categorizing individuals into either subgroup. Collectively, our findings underscore the pivotal role of the FPN in regulating sustained attention, particularly in states of sleep deprivation.

By quantifying the magnitude of spontaneous blood-oxygen-level-dependent (BOLD) signals, fALFF offers a measure of regional spontaneous activity over time. Prior resting-state fMRI research has demonstrated a correlation between fALFF and behavioral performance, and it has even been employed to predict attentional states under sleep deprivation (16). A decline in fALFF within the right inferior parietal lobule following 24 h of sleep deprivation has been documented, as well as diminished amplitude of fluctuations in the bilateral dorsolateral prefrontal cortex after 36 h of sleep deprivation (17, 18). These declines in fronto-parietal network activity echo findings from other neuroimaging studies examining the effects of acute sleep deprivation on sustained attention. For example, a PET-CT study showed significant metabolism reductions mainly in the frontal and parietal gyrus regions (19). De Havas et al. (20) observed reduced anti-correlation between the fronto-parietal network and the default mode network (DMN) using a seed-based approach. Additionally, Yeo et al. (15) classified participants into SD-resistant and SD-vulnerable groups and found that the resilient subjects displayed stronger anti-correlations between the FPN and DMN. Lastly, a recent study highlighted that the interplay between FPN and DMN activity influences cognitive performance, especially during the biological night (21).

The significance of the FPN in sustaining attention during sleep deprivation is further underscored by its association with the superior longitudinal fasciculus (SLF), a key association fiber tract linking the frontal and parietal lobes (22), prior research has revealed that individual differences in attentional lapses correlate with the microstructural properties of the SLF (23). In fact, greater white matter integrity facilitates efficient signal communication between frontal and parietal regions, potentially conferring enhanced resistance to sleep deprivation (24). Recent studies further affirm that the integrity of the SLF, along with the topological efficiency of the white matter network that includes frontal and parietal tracts, plays a pivotal role in individual variability in sleep deprivation resistance (14, 24). Moreover, research focusing on mild cognitive impairment (MCI) has also suggested that elevated FPN activity is linked with both improved sleep quality and enhanced cognitive performance (25).

Individual differences in sustained attention appear to be trait-like, as evidenced by the stability of SD-induced variations in PVT performance over time, even when assessments occur months or years apart (9). This vulnerability to SD is consistent with a heightened susceptibility to sleep restriction. A twin study further reinforced the idea that vigilance impairments due to SD are highly heritable (26). Consistent with existing literature indicating the prefrontal cortex’s vulnerability to SD, our study found that the SD-resistant group exhibited greater spontaneous activity in the left superior frontal gyrus. The prefrontal cortex is a central hub for various cognitive processes, notably vigilant regulation and top-down attentional control (27). Several investigations have demonstrated that baseline fALFF values in specific brain regions, including the DLPFC, are associated with cognitive performance and the susceptibility to sleep deprivation-induced impairments. These studies have often highlighted that individuals with higher baseline fALFF values in frontal regions tend to exhibit better cognitive performance and resilience to sleep deprivation-related deficits. For example, studies by Chee and (28) and Weissman et al. (11) have reported that individuals resistant to sleep deprivation display elevated activation levels within the frontoparietal network during sustained attention tasks compared to those who are more vulnerable. These findings align with our observation that the SD-resistant group exhibited higher spontaneous activity within the left DLPFC. The greater attentional deficit observed in the SD-vulnerable group could potentially be attributed to diminished top-down processing.

In light of our findings highlighting the pivotal role of the frontoparietal network (FPN) in sustained attention amidst sleep deprivation, it becomes imperative to speculate on the potential mechanisms underpinning these observations. The FPN is renowned for its integral role in high-order cognitive functions, encapsulating attention, working memory, and cognitive control. The perturbations in neural activity within this network during sleep deprivation might be indicative of the brain’s compensatory adaptations, striving to preserve cognitive performance despite the deficit in sleep. Biological undercurrents, such as fluctuations in neurotransmitter systems—specifically, adenosine, dopamine, and orexin—may offer an explanation for these neural modulations, as these are known to influence cognitive trajectories and have a pronounced impact during periods of sleep loss. Furthermore, the dynamics of intrinsic functional connectivity within the FPN, potentially modulated by external task demands and environmental contingencies, could also contribute to these alterations. While our study sheds light on the neural correlates of sleep deprivation and its impact on attention, it also uncovers a critical avenue for future research. Subsequent investigations should aspire to unravel these intricate mechanisms through a multi-modal imaging approach, integrating rs-fMRI with other methodologies like PET or EEG. Such endeavors would not only elucidate the complex neurobiological processes sustaining attention during sleep deprivation but also pioneer interventions to mitigate the cognitive detriments associated with sleep deficits. The findings from our study have substantial implications for strategies aimed at cognitive enhancement, particularly in scenarios that demand extended periods of wakefulness such as shift work, medical professions, military operations, and long-haul transportation. Understanding the neural underpinnings of sustained attention and the individual susceptibility to sleep deprivation opens avenues for personalized interventions to mitigate the cognitive deficits associated with sleep loss. For instance, individuals identified as vulnerable to sleep deprivation could benefit from tailored strategies such as scheduled napping, strategic caffeine use, or light therapy to enhance alertness. Moreover, neurofeedback and cognitive training could potentially strengthen the frontoparietal network, thereby improving resilience to sleep loss-related attentional lapses. It’s also conceivable that identifying genetic markers or trait-related predictors of sleep deprivation vulnerability could inform more targeted approaches for cognitive enhancement.

Several limitations should be considered. First, the study’s limited sample size calls for future research with larger cohorts to validate our conclusions. Second, although participants were instructed to remain awake and confirmed their alertness via post-scan self-reports, the potential occurrence of micro-sleeps during fMRI scanning cannot be entirely ruled out. To address this limitation, future studies could employ electroencephalography monitoring or eye-tracking technology for more definitive results. Additionally, while our study classified participants as SD-vulnerable or SD-resilient based on their PVT performance, a deeper analysis incorporating genomic data or psychological assessments could provide a more comprehensive view of the factors underlying this variability. Such an approach would require a multidisciplinary effort, combining neuroimaging with genomics, chronobiology, and psychology, to unravel the complex interplay of factors that determine individual sleep deprivation responses. Lastly, while our study did not include a longitudinal follow-up, we recognize its importance in elucidating the temporal dynamics of sleep deprivation effects on brain function and sustained attention. We advocate for subsequent research to explore these aspects, contributing to a more nuanced understanding of sleep deprivation consequences on cognitive health and informing strategies for recovery and intervention.

In summary, our study reveals that 24 h sleep deprivation leads to diminished spontaneous activity within the fronto-parietal network, particularly affecting various frontal and parietal gyri. These reductions in fALFF were significantly correlated with increased reaction times and a higher number of attentional lapses in the psychomotor vigilance task. Importantly, our findings suggest that baseline fALFF values in the left dorsolateral prefrontal cortex could serve as potential indicators for susceptibility to sleep deprivation-induced impairments in sustained attention. These results enrich the existing body of evidence on the critical role the FPN plays in attentional deficits stemming from sleep deprivation.

## Data availability statement

The raw data supporting the conclusions of this article will be made available by the authors, without undue reservation.

## Ethics statement

The studies involving humans were approved by Ethics Committee of Xi’an No. 9 Hospital. The studies were conducted in accordance with the local legislation and institutional requirements. The participants provided their written informed consent to participate in this study.

## Author contributions

LY: Methodology, Software, Writing – review & editing. YW: Investigation, Project administration, Resources, Writing – original draft. YG: Conceptualization, Validation, Visualization, Writing – review & editing. HG: Formal analysis, Supervision, Writing – review & editing. XG: Data curation, Visualization, Writing – original draft.
